# Nesfatin-1 levels and non-suicidal self-injury in adolescents with major depressive disorder: a cross-sectional study

**DOI:** 10.1186/s12888-026-08612-x

**Published:** 2026-09-09

**Authors:** Bedia Sultan Onal, Hilmi Furkan Arslan, Cansu Cobanoglu Osmanlı, Berkan Sahin, Gülsüm Yitik Tonkaz

**Affiliations:** 1 Department of Child and Adolescent Psychiatry, Faculty of Medicine, Giresun University, Giresun, Turkey; 2 Department of Medical Biochemistry, Faculty of Medicine, Giresun University, Giresun, Turkey

**Keywords:** Non-suicidal self-injury, Nesfatin-1, Depression, Adolescents, Neurobiological markers

## Abstract

**Background:**

Non-suicidal self-injury (NSSI) is a deliberate, repetitive act resulting in tissue damage without suicidal intent and is frequently associated with depressive disorders. Nesfatin-1, an anorexigenic peptide expressed in the hypothalamus, has been implicated in stress-related psychopathologies beyond its metabolic functions. This study aimed to investigate the relationship between serum nesfatin-1 levels and non-suicidal self-injury in adolescents diagnosed with major depressive disorder (MDD).

**Methods:**

This study included 40 adolescents aged 12–18 years with MDD, 41 adolescents with MDD and comorbid NSSI, and 45 healthy controls. All participants were medication-free for at least eight weeks prior to enrollment. Psychiatric diagnoses were established using K-SADS-PL. Depressive symptom severity and self-injurious behaviors were assessed using the CDI and ISAS, respectively. Serum nesfatin-1 levels were measured using a sandwich enzyme-linked immunosorbent assay (ELISA).

**Results:**

Serum nesfatin-1 levels differed significantly among the three groups (*p* < 0.001) and were highest in the MDD+NSSI group. Both patient groups exhibited significantly higher nesfatin-1 levels than healthy controls, and the MDD+NSSI group showed significantly higher levels than the MDD group (*p* = 0.010), despite comparable depressive symptom severity between the two patient groups. Serum nesfatin-1 levels were positively correlated with depressive symptom severity (Spearman’s ρ = 0.691, *p* < 0.001). ROC curve analyses demonstrated good discriminative ability of nesfatin-1 for distinguishing patient groups from healthy controls and fair discriminative ability for differentiating adolescents with MDD+NSSI from those with MDD alone (AUC = 0.671, 95% CI: 0.553–0.789, *p* = 0.008). CDI scores did not differ between the two patient groups (*p* = 0.059) despite both being significantly higher than those of the control group (*p* < 0.001).

**Conclusions:**

Serum nesfatin-1 levels were associated with both depressive symptom severity and NSSI in adolescents with MDD. Notably, nesfatin-1 levels differentiated adolescents with MDD+NSSI from those with MDD alone despite comparable depressive symptom severity between the two patient groups. These findings suggest that nesfatin-1 may be involved in neurobiological processes underlying self-injurious behavior in adolescent depression. Future longitudinal studies with larger samples are needed to further clarify the role and clinical relevance of nesfatin-1 in adolescents with depression and NSSI.

**Trial registration:**

Not applicable.

## Background

Non-suicidal self-injury (NSSI) is defined as the deliberate and repetitive destruction of one’s own body tissue without suicidal intent [1]. In DSM-5, NSSI was introduced as a distinct clinical condition under “Other Conditions That May Be a Focus of Clinical Attention” [2]. NSSI is typically performed to reduce negative affect, resolve interpersonal difficulties, or induce a desired emotional state [3]. Although the prevalence of specific NSSI methods varies across studies and populations, self-hitting, interfering with wound healing, and cutting are among the most commonly reported behaviors in adolescent samples [4, 5]. The majority of adolescents engaging in NSSI report using multiple methods of self-injury [4].

The etiology of self-injurious behavior involves multiple psychosocial, genetic, and neurobiological factors [6]. Prevalence studies have reported rates ranging between 18% and 23% in adolescents, and a meta-analysis of approximately 600,000 adolescents aged 12–18 years from 41 countries reported a lifetime prevalence of 16.9%, with an increasing trend over time [7, 8]. NSSI frequently co-occurs with psychiatric disorders, particularly depression, as well as anxiety disorders, eating disorders, behavioral disorders, and alcohol/substance use disorders [3, 9]. Depression has been identified as the most common psychiatric condition associated with NSSI. Despite the well-established association between depression and NSSI, the neurobiological mechanisms underlying this comorbidity remain poorly understood. Nesfatin-1, a neuropeptide implicated in stress regulation and mood disorders, represents a potentially relevant candidate in this context.

Nesfatin-1 is an 82-amino acid anorexigenic neuropeptide derived from nucleobindin-2 (NUCB2) that is expressed predominantly in the hypothalamus and plays a role in food intake and energy regulation [10–12]. Beyond its metabolic functions, nesfatin-1 exerts significant effects on multiple neurotransmitter systems and neurocognitive processes, and accumulating evidence indicates that nesfatin-1 levels are altered in various stress-related psychopathologies [13, 14].

Experimental studies in rodents have demonstrated that intracerebroventricular administration of nesfatin-1 activates the hypothalamic–pituitary–adrenal (HPA) axis and increases depressive-like behavior, anxiety-like behavior, and anhedonia [15]. Consistent with these findings, clinical studies in adults and adolescents have generally reported elevated nesfatin-1 levels in individuals with major depressive disorder, together with positive associations between nesfatin-1 levels and depression severity [16–19], although findings have not been entirely consistent, as reduced levels have also been reported in depressed adolescents [20]. Nesfatin-1 has additionally been examined in relation to suicidal ideation, with studies suggesting that it may serve as a biomarker of depression severity and suicide risk [20–22].

Although NSSI is a well-established predictor of future suicidal behavior and its prevalence during adolescence has increased considerably in recent years [23, 24], it is increasingly recognized as a distinct clinical condition with neurobiological characteristics that may not fully overlap with those of depression or suicidality. Growing evidence suggests that neurobiological mechanisms related to stress and affect regulation may contribute to the development and maintenance of NSSI [25, 26]. Given the proposed involvement of nesfatin-1 in stress regulation, mood modulation, and HPA axis activity, examining nesfatin-1 specifically in adolescents with NSSI may clarify whether this neuropeptide is associated with self-injurious behavior beyond depressive psychopathology. To date, however, studies have examined nesfatin-1 only in relation to depression or suicide probability [19, 20], and no study has specifically investigated nesfatin-1 levels in depressed adolescents with NSSI. Therefore, the present study aimed to compare serum nesfatin-1 levels among adolescents with MDD with and without NSSI and healthy controls, and to examine the association between serum nesfatin-1 levels and depressive symptom severity.

The study hypotheses were as follows: [1] serum nesfatin-1 levels would be higher in adolescents with MDD than in healthy controls; [2] serum nesfatin-1 levels would differ between adolescents with MDD with and without NSSI; and [3] serum nesfatin-1 levels would be positively correlated with depressive symptom severity.

## Methods

### Study design and setting

This cross-sectional study was conducted at the Giresun Maternity and Pediatrics Training and Research Hospital, child and adolescent psychiatry outpatient clinic. Participants were consecutively recruited following eligibility assessment based on predefined inclusion and exclusion criteria. Written informed consent was obtained from all participants and their legal guardians prior to enrollment.

A total of 357 adolescents presenting with depressive complaints were initially assessed for eligibility. Of these, 261 were excluded based on the predefined exclusion criteria, and 15 were additionally excluded due to a history of suicide attempt. The final patient sample comprised 81 adolescents: 40 diagnosed with major depressive disorder (MDD group) and 41 diagnosed with major depressive disorder with comorbid non-suicidal self-injury (MDD+NSSI group).

**Fig. 1:**
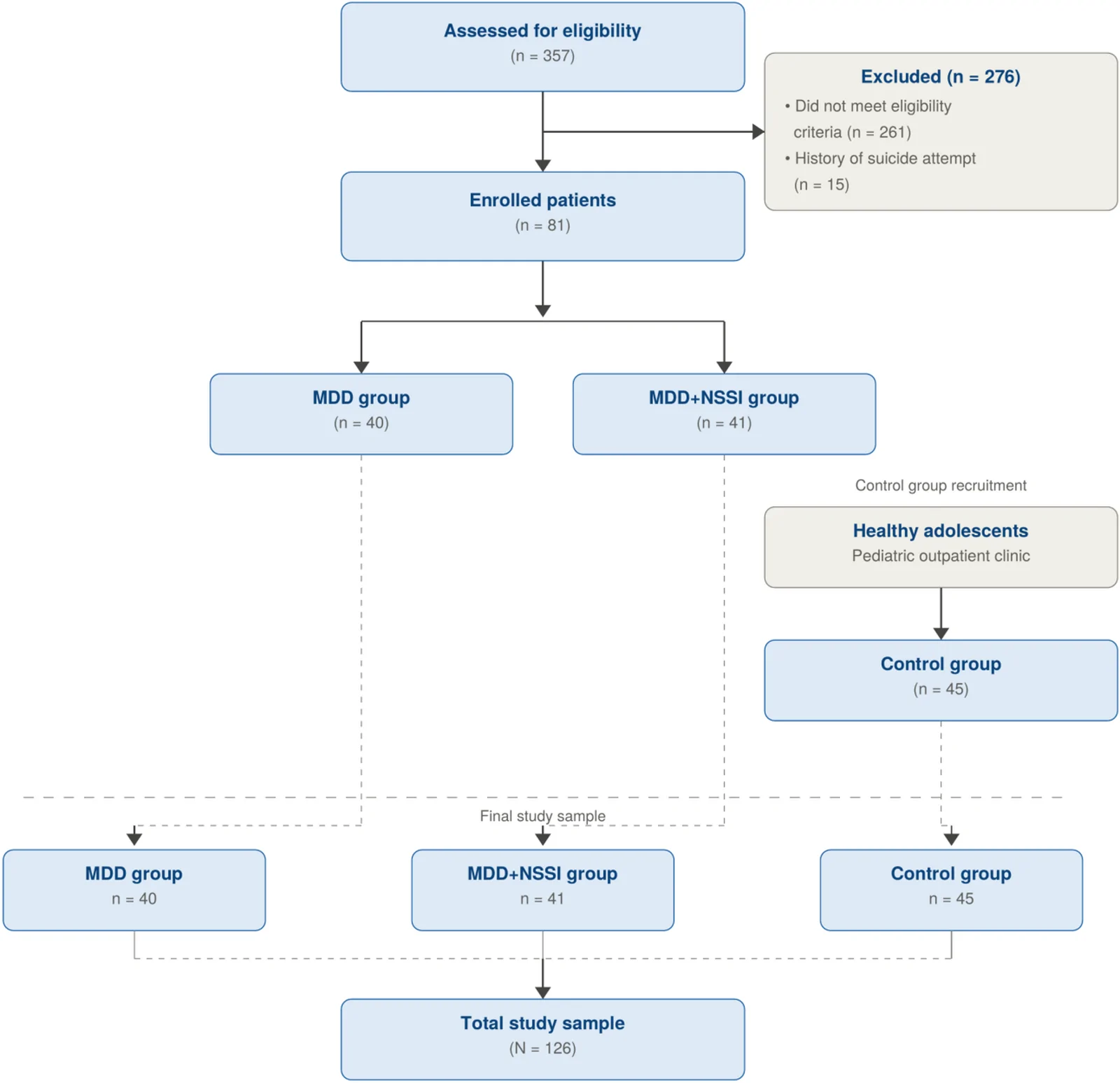
Flow diagram of participant recruitment and group allocation. *MDD: Major Depressive Disorder; NSSI: Non-Suicidal Self-Injury

The control group consisted of adolescents attending the pediatric outpatient clinics of the same hospital for routine well-child visits. The control participants had no history of psychiatric disorders, met all eligibility criteria, and were matched to the patient groups by age and sex. A total of 45 healthy adolescents were enrolled, resulting in an overall study sample of 126 participants. The participant recruitment and group allocation flow diagram is presented in Fig. 1.

### Participants

#### Inclusion criteria

Participants in the MDD and MDD+NSSI groups were required to meet the following criteria: [1] age between 12 and 18 years; [2] a DSM-5 diagnosis of major depressive disorder confirmed by a child and adolescent psychiatrist using the K-SADS-PL, without (MDD group) or with (MDD+NSSI group) comorbid non-suicidal self-injury; [3] no prior history of suicide attempt; and [4] written informed consent provided by both the participant and their legal guardian following a detailed explanation of the study procedures. Participants in the control group were required to: [1] be aged between 12 and 18 years; [2] have no known chronic medical illness or psychiatric disorder, as confirmed by clinical assessment; [3] have no history of non-suicidal self-injury or suicide attempt; and [4] provide written informed consent by both the participant and their legal guardian.

#### Exclusion criteria

Participants in the MDD and MDD+NSSI groups were excluded if they had: [1] a comorbid psychiatric disorder, including autism spectrum disorder, intellectual disability, attention-deficit/hyperactivity disorder, bipolar disorder, psychotic disorders; [2] psychotropic or other medication use within the eight weeks prior to enrollment; [3] a chronic medical condition, including hypertension, hypothyroidism, renal disease, cardiac disease, neurological disorders, active infection, malignancy, or dermatological disease; [4] metabolic syndrome, obesity, diabetes mellitus, or other endocrine disorders potentially affecting nesfatin-1 levels; [5] recent surgical procedures or physical trauma; [6] tobacco, alcohol, or substance use; [7] inability to obtain written informed consent. Control participants were excluded if they had any psychiatric disorder or chronic medical illness; a history of tobacco, alcohol, or substance use, non-suicidal self-injury; suicide attempt; or inability to obtain written informed consent.

### Sample size calculation

The sample size was determined based on previous studies using a G*Power a priori power analysis [16, 20]. The effect size (f = 0.388) was informed by the findings of a previous study examining nesfatin-1 levels in adolescents with major depressive disorder [20]. A one-way ANOVA design with three groups, an effect size of f = 0.388, a significance level of α = 0.05, and a statistical power of 0.95 yielded a minimum required sample size of 36 participants per group (total *n* = 108). The final sample of 126 participants exceeded this minimum requirement.

### Data collection procedure

Following the psychiatric examination of adolescents aged 12–18 years who presented to the Child and Adolescent Psychiatry Clinic of Giresun Maternity and Children Training and Research Hospital, a semi-structured diagnostic interview, the Schedule for Affective Disorders and Schizophrenia for School-Age Children – Present and Lifetime Version (K-SADS-PL), was conducted by a child and adolescent psychiatry specialist to establish psychiatric diagnoses. During the clinical interview, sociodemographic information was obtained from the participants, and data collection forms were completed by the researchers. At the initial visit, participants completed the study procedures, which included diagnostic interviews, completion of self-report scales, and biochemical assessments. The participants completed the Children’s Depression Inventory and the Inventory of Statements About Self-Injury independently, and a researcher was present during scale administration to clarify any items that were not understood.

### Laboratory measurements

In routine clinical practice, patients for whom treatment is planned to follow a diagnosis of depression undergo standard biochemical and hematological testing to exclude organic causes that may lead to depressive-like symptoms, such as vitamin deficiencies, anemia, or hypothyroidism. In the present study, following the completion of psychiatric evaluations, blood samples were obtained simultaneously for the assessment of serum nesfatin-1 levels in addition to routine biochemical and hematological tests. In the control group, serum nesfatin-1 samples were collected concurrently with routine laboratory tests performed during scheduled healthy child follow-up visits at pediatric outpatient clinics. No additional venipuncture was performed solely for the purpose of measuring nesfatin-1 in either the patient or control groups. For the measurement of serum nesfatin-1 levels, venous blood samples were collected from the antecubital vein between 8:30 and 10:30 a.m. following an overnight fast of 8–12 h. Blood samples were carefully and immediately transferred from collection tubes into centrifuge tubes, and serum was separated and stored at − 80 °C until analysis. Routine biochemical and hematological tests were completed on the same day. Serum nesfatin-1 levels were measured using a commercially available sandwich enzyme-linked immunosorbent assay (ELISA) kit (Elabscience Human NES1–Nesfatin-1 ELISA Kit). Serum nesfatin-1 concentrations were reported in picograms per milliliter (pg/mL). The samples obtained from the patient groups were analyzed in duplicate.

### Instruments

#### Data collection form

The data collection form was developed by the researchers and included items assessing participants’ sociodemographic characteristics (age, sex, educational level, family structure, parental education and occupational status, and socioeconomic status) as well as clinical characteristics.

#### Schedule for affective disorders and schizophrenia for school-age children – present and lifetime version (K-SADS-PL)

The K-SADS-PL is a semi-structured diagnostic interview developed to assess current and lifetime psychopathology in children and adolescents [27]. The validity and reliability of the Turkish version were established by Gökler et al. [28].

#### Children’s Depression Inventory (CDI)

The Children’s Depression Inventory (CDI) is a self-report scale used to assess depressive symptoms in children aged 6–17 years and was adapted into Turkish by Oy [29, 30]. The CDI consists of 27 items, each with three response options. Participants are asked to select the statement that best describes their feelings over the past two weeks. Each item is scored as 0, 1, or 2 according to symptom severity, yielding a maximum total score of 54. Higher scores indicate greater severity of depressive symptoms. A cutoff score of 19 is recommended.

#### Inventory of statements about self-injury (ISAS)

The Inventory of Statements About Self-Injury (ISAS) is a two-part instrument designed to assess non-suicidal self-injury (NSSI). Section I evaluates the lifetime frequency of 12 types of self-injurious behaviors [31]. Section II consists of 39 items assessing 13 distinct functions of NSSI across two higher-order dimensions: intrapersonal functions (affect regulation, suicide prevention, labeling distress, self-punishment, and anti-dissociation) and interpersonal functions (interpersonal boundaries, interpersonal influence, revenge, sensation seeking, peer bonding, toughness, autonomy, and self-care) [32]. Each item is rated on a three-point Likert scale ranging from 0 (“not relevant”) to 2 (“very relevant”). The Turkish validity and reliability study was conducted by Bildik et al. [33].

### Statistical analysis

Statistical analyses were performed using the Statistical Package for the Social Sciences (SPSS) software (version 25; IBM Corp., Armonk, NY, USA). The normality of continuous variables was assessed using the Shapiro–Wilk test and visual inspection of histograms. Descriptive statistics were expressed as mean ± standard deviation for continuous variables and as frequencies and percentages for categorical variables. Comparisons of sociodemographic and clinical variables among the three groups were conducted using one-way analysis of variance (ANOVA) for continuous variables and Fisher’s exact test for categorical variables. When a significant main effect was detected in ANOVA, Tukey’s post hoc test was applied for pairwise group comparisons. Serum nesfatin-1 levels and Children’s Depression Inventory (CDI) scores were compared across groups using one-way ANOVA followed by Tukey post hoc analyses. The associations between serum nesfatin-1 levels and CDI scores, as well as ISAS Section II functional scores, were examined using Spearman’s rank correlation coefficient. To assess the robustness of the primary parametric analyses, supplementary non-parametric sensitivity analyses were performed using the Kruskal–Wallis test followed by pairwise Mann–Whitney U tests with Bonferroni correction (adjusted significance threshold: *p* < 0.017). An additional analysis of covariance (ANCOVA) was performed to examine whether between-group differences in serum nesfatin-1 levels remained after adjustment for depressive symptom severity. Serum nesfatin-1 level was entered as the dependent variable, study group as the fixed factor, and CDI score as a covariate. Bonferroni-adjusted p values were used for pairwise comparisons. Prior to ROC curve analysis, nesfatin-1 values were screened for outliers across all three groups using boxplot inspection. Five extreme values identified in the control group were excluded, resulting in a final control group sample of 40 participants for the ROC analyses. No extreme outliers were identified in either the MDD or the MDD+NSSI group. Receiver operating characteristic (ROC) curve analysis was performed to evaluate the diagnostic utility of serum nesfatin-1 levels in discriminating between groups. The area under the curve (AUC), sensitivity, specificity, and optimal cut-off values were determined. The optimal cut-off point was identified using the Youden Index (sensitivity + specificity − 1). Statistical significance was defined as a two-tailed p value < 0.05, except for Bonferroni-corrected pairwise Mann–Whitney U comparisons, for which *p* < 0.017 was considered significant.

## Results

The study sample consisted of 126 adolescents. Among these, 40 adolescents (6 males, 34 females) were diagnosed with MDD, 41 adolescents (7 males, 34 females) were diagnosed with MDD+NSSI, and 45 adolescents (11 males, 34 females) constituted the healthy control group. There were no significant differences between the patient and control groups in terms of sex, educational status, maternal and paternal education levels, family structure, family socioeconomic status, or place of residence (Table 1).

There were no significant differences between the patient and control groups in terms of age, maternal age, paternal age, or body mass index. The mean body mass index was 21.44 ± 3.81 in the group diagnosed with major depressive disorder, 21.95 ± 4.12 in the group with major depressive disorder and non-suicidal self-injury, and 21.81 ± 3.26 in the healthy control group. Comparisons of age, maternal age, paternal age, and body mass index are presented in Table 1.

**Table 1:** Sociodemographic and clinical characteristics of the study groups

Variables	MDD	MDD+NSSI	Control	Statistics	*p*
	(*n* = 40)	(*n* = 41)	(*n* = 45)		
Sex, n (%)					
Female	34 (85)	34 (82.9)	34 (75.6)	Fisher	0.557
Male	6 (15)	7 (17.1)	11 (24.4)		
Educational status, n (%)					
Not attending	2 (5)	5 (12.2)	2 (4.4)	Fisher	0.709
8th grade	7 (17.5)	4 (9.8)	9 (20)		
9th grade	7 (17.5)	8 (19.5)	9 (20)		
10th grade	12 (30)	6 (14.6)	8 (17.8)		
11th grade	5 (12.5)	7 (17.1)	8 (17.8)		
12th grade	7 (17.5)	11 (26.8)	9 (20)		
Mother’s educational status, n (%)					
Literate	0 (0)	1 (2.5)	0 (0)	Fisher	0.128
Primary school	26 (65)	22 (55)	17 (37.8)		
High school	12 (30)	13 (32.5)	22 (48.9)		
University or above	2 (5)	4 (10)	6 (13.3)		
Father’s educational status, n (%)					
Literate	0 (0)	0 (0)	0 (0)	Fisher	0.403
Primary school	25 (64.1)	28 (68.3)	22 (48.9)		
High school	10 (25.6)	8 (19.5)	16 (35.6)		
University or above	4 (10.3)	5 (12.2)	7 (15.6)		
Family structure					
Nuclear family	30 (75)	25 (61)	36 (80)	Fisher	0.179
Extended family	7 (17.5)	7 (17.1)	7 (15.6)		
Separated/divorced parents	2 (5)	8 (19.5)	2 (4.4)		
Parental loss	1 (2.5)	1 (2.4)	0 (0)		
Socioeconomic status, n (%)					
Class I (upper class)	0 (0)	0 (0)	0 (0)	Fisher	0.152
Class II (upper-middle class)	2 (5)	4 (9.8)	4 (8.9)		
Class III (middle class)	12 (30)	9 (22)	17 (37.8)		
Class IV (lower-middle class)	13 (32.5)	14 (34.1)	18 (40)		
Class V (lower class)	12 (30)	9 (22)	6 (13.3)		
Unknown	1 (2.5)	5 (12.2)	0 (0)		
Place of residence, n (%)					
Urban (city center)	20 (50)	19 (46.3)	29 (64.4)	Fisher	0.43
District town	17 (42.5)	18 (43.9)	12 (26.7)		
Rural/village	3 (7.5)	4 (9.8)	4 (8.9)		
Age (years), Mean ± SD	15.10 ± 1.46	15.39 ± 1.51	15.00 ± 1.58	F = 0.748	0.476
BMI (kg/m²), Mean ± SD	21.44 ± 3.81	21.95 ± 4.12	21.81 ± 3.26	F = 0.195	0.823
Mother’s age, Mean ± SD	45.05 ± 5.83	43.28 ± 6.07	42.29 ± 5.87	F = 2.341	0.101
Father’s age, Mean ± SD	49.59 ± 6.41	47.05 ± 6.02	47.02 ± 6.67	F = 2.122	0.124

*Categorical variables were compared using Fisher’s exact test due to small expected cell counts. Continuous variables were compared using one-way ANOVA. A two-tailed p value < 0.05 was considered statistically significant. Data on maternal and paternal educational status were missing for one participant (*n* = 125). Socioeconomic status was assessed using the Hollingshead–Redlich Scale (Class I: highest; Class V: lowest). BMI: Body Mass Index; MDD: Major Depressive Disorder; NSSI: Non-Suicidal Self-Injury; SD: Standard deviation

The mean CDI score was 28.03 ± 5.85 in the MDD group, 31.15 ± 7.52 in the MDD+NSSI group, and 7.82 ± 4.68 in the healthy control group. One-way analysis of variance (ANOVA) performed on CDI scores revealed a significant difference among the three groups (F = 187.838, *p* < 0.001). The highest mean CDI score was observed in the MDD+NSSI group, while the lowest mean CDI score was observed in the healthy control group (Table 2).

According to the Tukey post hoc analysis, CDI scores were significantly higher in both the MDD group and the MDD+NSSI group compared to the healthy control group (*p* < 0.001 for both comparisons). However, no significant difference was found between the two patient groups, namely the MDD group and the MDD+NSSI group (*p* = 0.059) (Table 2).

**Table 2:** Comparison of CDI scores among the study groups

	MDD	MDD+NSSI	Control		
	Mean ± SD	Mean ± SD	Mean ± SD	F	p
**CDI scores**	28.03 ± 5.85^a^	31.15 ± 7.52^a^	7.82 ± 4.68^b^	187.838	**< 0.001**

*Between-group comparisons were performed using one-way analysis of variance (ANOVA), followed by Tukey’s honestly significant difference (HSD) post hoc test. Groups sharing the same superscript letter do not differ significantly from each other. Groups with different superscript letters differ significantly (*p* < 0.05). CDI: Children’s Depression Inventory; MDD: Major Depressive Disorder; NSSI: Non-Suicidal Self-Injury; SD: Standard deviation

In the MDD+NSSI group, the most frequently reported method of self-injury was hitting oneself or banging against objects (73.2%). This was followed by interfering with wound healing (68.3%), pinching (48.8%), and scratching (48.8%). The mean number of self-injury methods used was 5.32 ± 2.89, and 95.1% of participants reported using more than one method of non-suicidal self-injury. The least frequently reported methods were ingesting or swallowing substances (19.5%) and behaviors categorized as “other” (4.9%) (Table 3). The mean age at onset of non-suicidal self-injury was 12.78 ± 1.57 years, and the time since the most recent NSSI episode was reported as 3.15 ± 3.15 weeks. In the MDD+NSSI group, the mean intrapersonal function score was 14.68 ± 6.77, the mean Interpersonal function score was 11.83 ± 8.01, and the mean total ISAS function score was 26.46 ± 13.43.

The mean serum nesfatin-1 level was 526.44 ± 296.17 pg/mL in patients with MDD, 688.61 ± 299.12 pg/mL in the MDD+NSSI group, and 98.72 ± 92.95 pg/mL in the healthy control group. The difference in mean serum nesfatin-1 levels among the groups was statistically significant (F = 67.093, *p* < 0.001). The highest mean nesfatin-1 level was observed in the MDD+NSSI group, while the lowest mean nesfatin-1 level was observed in the healthy control group (Table 4). These findings were confirmed by non-parametric sensitivity analyses. Serum nesfatin-1 levels differed significantly across the three groups (Kruskal–Wallis H = 80.833, *p* < 0.001), and pairwise Mann–Whitney U comparisons confirmed significantly higher nesfatin-1 levels in the MDD+NSSI group than in the MDD group (Z = − 2.631, *p* = 0.009), which remained significant after Bonferroni correction. Consistent with the primary analysis, CDI scores did not differ significantly between the two patient groups (Z = − 1.807, *p* = 0.071).

**Table 3:** Characteristics and functional dimensions of non-suicidal self-injury behaviors assessed with the ISAS

Variables	Prevalence	Frequency
	*n* (%)	Mean ± SD
NSSI Methods		
Banging/Hitting self	30(73.2)	21.54 ± 32.79
Interfering with wound healing (e.g., picking scabs)	28(68.3)	36.76 ± 42.49
Cutting	27(65.9)	11.54 ± 25.84
Scratching	20(48.8)	17.41 ± 33.63
Pinching	20(48.8)	11.68 ± 24.41
Biting	19(46.3)	10.56 ± 26.15
Pulling hair	17(41.5)	10.39 ± 23.11
Rubbing skin against rough surface	15(36.6)	10.37 ± 26.69
Carving	13(31.7)	3.68 ± 15.63
Sticking self with needles	12(29.3)	6.78 ± 21.82
Swallowing dangerous substances	8(19.5)	0.54 ± 1.47
Burning	7(17.1)	1.88 ± 8.02
Other	2(4.9)	0.24 ± 1.10
> 1 NSSI types	39(95.1)	
Numbers of NSSI methods		5.32 ± 2.89
Age at NSSI onset (years)		12.78 ± 1.57
Time since last NSSI (weeks)		3.15 ± 3.15
**ISAS Section II**		
Intrapersonal function score		14.68 ± 6.77
Interpersonal function score		11.83 ± 8.01
Total function score		26.46 ± 13.43

*Data are presented for the MDD+NSSI group only (*n* = 41). Methods are presented in descending order of prevalence. Values are expressed as mean ± standard deviation, or n (%). ISAS: Inventory of Statements About Self-Injury; MDD+NSSI: Major Depressive Disorder with Non-Suicidal Self-Injury; SD: Standard deviation

**Table 4:** Comparison of nesfatin-1 levels among the study groups

	MDD	MDD+NSSI	Control	F	*p*
	Mean ± SD	Mean ± SD	Mean ± SD		
**Nesfatin-1 pg/mL**	526.44 ± 296.17^a^	688.61 ± 299.12^b^	98.72 ± 92.95^c^	67.093	**< 0.001**

*Between-group comparisons were performed using one-way analysis of variance (ANOVA), followed by Tukey’s honestly significant difference (HSD) post hoc test. Groups sharing the same superscript letter do not differ significantly from each other. Groups with different superscript letters differ significantly (*p* < 0.05). MDD: Major Depressive Disorder; NSSI: Non-Suicidal Self-Injury; SD: Standard deviation

According to the Tukey post hoc analysis, serum nesfatin-1 levels were significantly higher in both the MDD group and the MDD+NSSI group compared to the healthy control group (*p* < 0.001 for both comparisons). In addition, a significant difference was also observed between the MDD group and the MDD+NSSI group (*p* = 0.010).

**Fig. 2:**
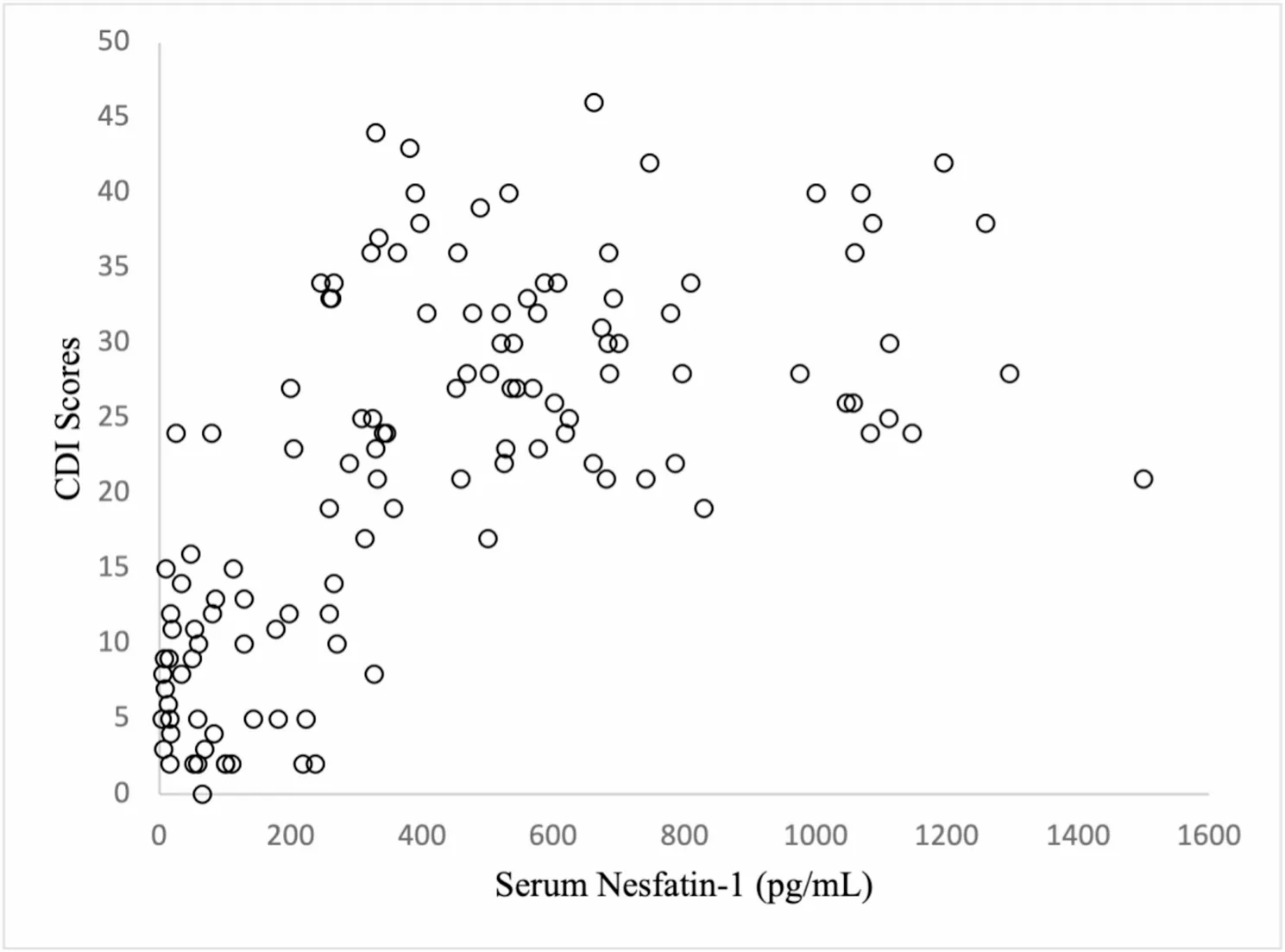
Scatter plot showing the correlation between serum nesfatin-1 levels and CDI scores. * Spearman’s rank correlation analysis revealed a significant positive correlation between serum nesfatin-1 levels and CDI scores (ρ = 0.691, *p* < 0.001). CDI: Children’s Depression Inventory

A positive correlation was observed between serum nesfatin-1 levels and CDI scores (Spearman’s ρ = 0.691, *p* < 0.001) (Fig. 2), indicating that higher depressive symptom severity was associated with higher serum nesfatin-1 levels. Nesfatin-1 levels were not significantly correlated with ISAS Section II intrapersonal function scores (ρ = −0.044, *p* = 0.786), interpersonal function scores (ρ = −0.114, *p* = 0.478), or total function scores (ρ = −0.101, *p* = 0.531).

To determine whether the observed between-group differences in serum nesfatin-1 levels were independent of depressive symptom severity, an analysis of covariance (ANCOVA) was performed with CDI score entered as a covariate. The effect of group remained significant after adjustment for CDI score (F = 15.391, *p* < 0.001, partial η² = 0.201), whereas CDI score did not significantly contribute to the model (F = 0.183, *p* = 0.670). Bonferroni-adjusted pairwise comparisons showed that estimated marginal serum nesfatin-1 levels remained significantly higher in the MDD+NSSI group (674.11 pg/mL) than in the MDD group (516.80 pg/mL; *p* = 0.017), and both patient groups differed significantly from healthy controls (120.50 pg/mL; both *p* < 0.001).

To evaluate the diagnostic utility of nesfatin-1 in discriminating between groups, receiver operating characteristic (ROC) curve analyses were performed for each pairwise comparison. The area under the curve (AUC), optimal cut-off value (determined by the Youden Index), sensitivity, and specificity are presented in Table 5.

**Table 5:** ROC curve analysis results for nesfatin-1 in discriminating between groups

	MDD & Control	MDD+NSSI & Control	MDD+NSSI & MDD
AUC	0.848	0.891	0.671
95% CI	0.766–0.931	0.829–0.962	0.553–0.789
p value	< 0.001	< 0.001	0.008
Optimal cut-off (pg/mL)	225.09	281.28	593.87
Sensitivity	77.5%	82.5%	61.0%
Specificity	82.2%	84.4%	75.0%
Youden index	0.597	0.670	0.360

*AUC: Area Under the Curve; CI: Confidence Interval. Optimal cut-off determined by Youden Index (Sensitivity + Specificity – 1). Positive state defined as the disease group in each comparison

**Fig. 3:**
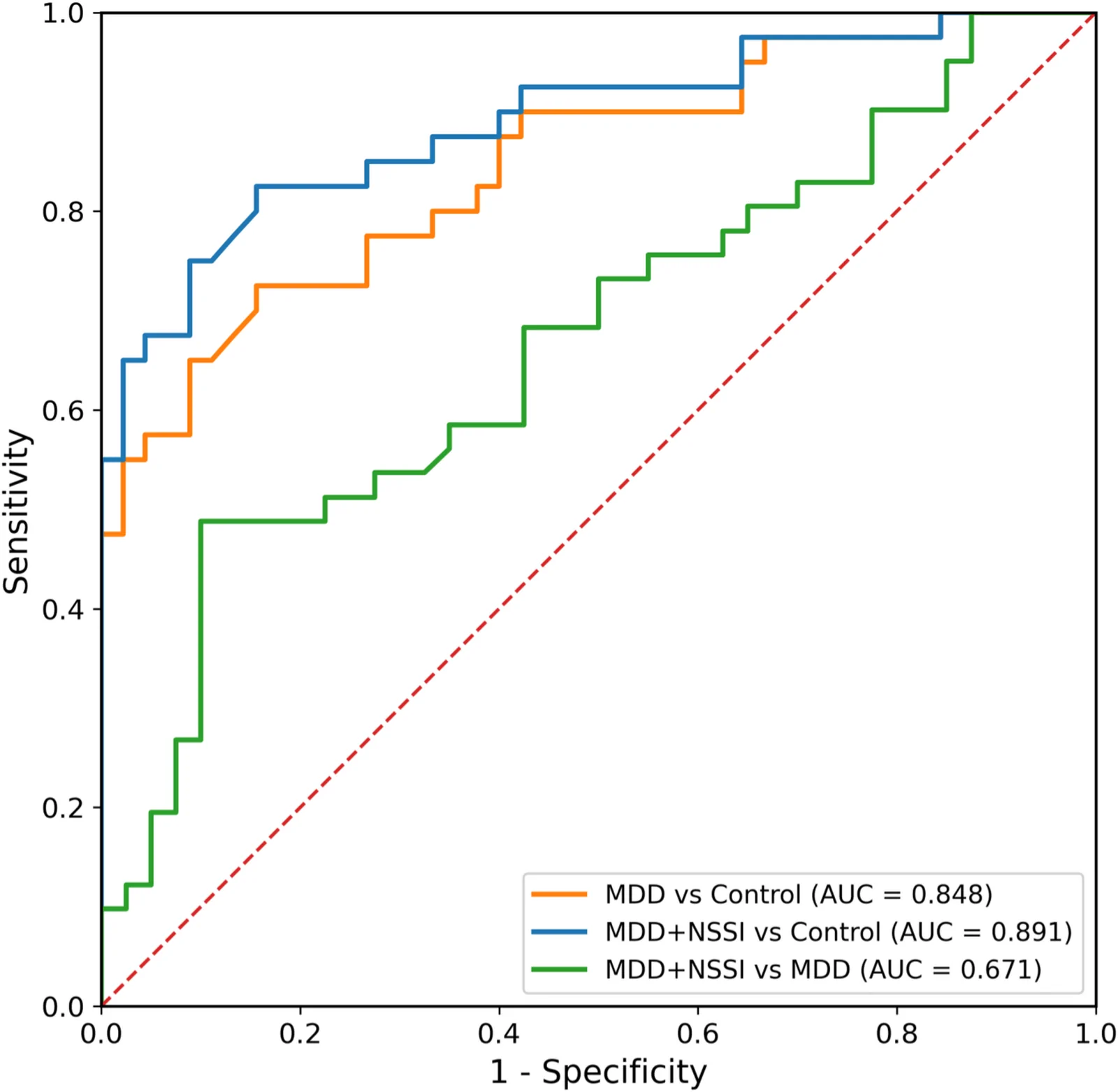
ROC curves of nesfatin-1 levels for discriminating between groups. *Receiver operating characteristic (ROC) curves of nesfatin-1 levels for discriminating between (1) MDD and controls (2), MDD+NSSI and controls, and (3) MDD+NSSI and MDD groups. Nesfatin-1 levels demonstrated good discriminative ability for MDD vs. control (AUC = 0.848) and MDD+NSSI vs. control (AUC = 0.891), while the discriminative performance between MDD+NSSI and MDD was fair (AUC = 0.671)

Nesfatin-1 demonstrated strong discriminative performance between the MDD group and healthy controls (AUC = 0.848, 95% CI: 0.766–0.931, *p* < 0.001), with an optimal cut-off of 225.09 pg/mL, a sensitivity of 77.5%, and a specificity of 82.2%. Similarly, nesfatin-1 showed excellent discriminative ability in distinguishing the MDD+NSSI group from healthy controls (AUC = 0.891, 95% CI: 0.829–0.962, *p* < 0.001), with an optimal cut-off of 281.28 pg/mL yielding a sensitivity of 82.5% and a specificity of 84.4%. When comparing the MDD+NSSI and MDD groups, nesfatin-1 exhibited statistically significant but fair discriminatory ability (AUC = 0.671, 95% CI: 0.553–0.789, *p* = 0.008), with an optimal cut-off value of 593.87 pg/mL, a sensitivity of 61.0%, and a specificity of 75.0%. ROC curves for all three comparisons are illustrated in Fig. 3.

## Discussion

This study is the first to investigate the association between serum nesfatin-1 levels and non-suicidal self-injury in adolescents with major depressive disorder. Our findings demonstrated that serum nesfatin-1 levels were significantly higher in depressed adolescents with NSSI compared with both adolescents with MDD without NSSI and healthy controls, and that nesfatin-1 levels were positively correlated with depressive symptom severity. Notably, although CDI scores did not differ significantly between the two patient groups, serum nesfatin-1 levels remained significantly higher in the MDD+NSSI group, suggesting that nesfatin-1 may reflect neurobiological differences associated with self-injurious behavior that extend beyond depressive symptom severity. The absence of significant sociodemographic differences between the groups further supports the comparability of the study sample.

NSSI and MDD share overlapping yet partially distinct neurobiological underpinnings, and their frequent co-occurrence in adolescence suggests the involvement of common pathophysiological mechanisms [24, 34]. Emerging neuroimaging evidence indicates that adolescents with NSSI and depression exhibit abnormalities in corticostriatal and frontolimbic networks involved in emotion regulation, reward processing, and stress responsivity [25, 26]. At the same time, NSSI is characterized by features that may partially distinguish it from depressive psychopathology alone, including dysregulation of the endogenous opioid system, with reduced plasma beta-endorphin levels reported in adolescents with NSSI [3]. Nesfatin-1, through its co-localization with stress-responsive neurons and its modulatory effects on HPA axis activity, represents a potentially relevant neuropeptide in this context. Sex-related biological differences may also be relevant when interpreting these findings. The predominance of female participants in the MDD+NSSI group is consistent with epidemiological evidence indicating a higher prevalence of NSSI among female adolescents [4, 24]. However, the small number of male participants precluded examination of potential sex-specific differences in nesfatin-1 levels.

The elevated serum nesfatin-1 levels observed in both patient groups may relate to the established role of nesfatin-1 in HPA axis regulation and stress responsivity. Nesfatin-1 is co-expressed with corticotropin-releasing hormone neurons in the paraventricular nucleus, and intracerebroventricular administration activates the HPA axis and increases depressive-like and anxiety-like behaviors in rodents [12, 14, 35]. Clinical findings further support this association, as nesfatin-1 levels have been positively correlated with corticosterone levels in patients with MDD [36]. Nesfatin-1 also interacts with serotonergic neurotransmission, as activation of hypothalamic 5-HT2C receptors has been associated with increased NUCB2/nesfatin-1 expression [16, 37]. Notably, experimental evidence suggests that nesfatin-1 responses may vary according to the duration and intensity of stress exposure, with acute stress increasing peripheral nesfatin-1 levels more consistently than chronic stress [14]. Taken together, these findings provide a possible stress-related biological context for the higher serum nesfatin-1 levels observed in the MDD+NSSI group. It should be emphasized, however, that HPA axis activity, serotonergic function, and endogenous opioid system activity were not directly assessed in the present study. These mechanistic interpretations therefore remain hypothetical, and no causal conclusions can be drawn from the present cross-sectional design.

Our findings are in line with a growing body of evidence demonstrating elevated nesfatin-1 levels in MDD. Studies in adults have consistently reported higher nesfatin-1 levels in patients with MDD compared with healthy controls, with positive associations between nesfatin-1 levels and depression severity [17, 18, 22, 38], and similar findings have been reported in adolescent samples from Turkey and China [16, 19]. Findings have not been entirely consistent, however. Karadeniz et al. [20] observed significantly lower serum nesfatin-1 levels in drug-naïve adolescents with first-episode MDD and found no significant association with depression severity, proposing that stress-related cortisol hypersecretion may suppress nesfatin-1 release through inhibitory feedback. Notably, that study did not distinguish adolescents according to the presence of NSSI, which may be relevant when comparing its findings with the present study. Additional variability across studies may arise from differences in ELISA methods, sample handling, illness stage, and sample characteristics. Interestingly, Korucu et al. [21] reported lower nesfatin-1 levels in adults with MDD and suicidal ideation, with a negative correlation between nesfatin-1 levels and suicidal ideation scores, raising the possibility that nesfatin-1 dysregulation may differ qualitatively between non-suicidal self-injury and suicidal ideation.

One of the most clinically relevant findings of the present study is the dissociation between CDI scores and serum nesfatin-1 levels across the two patient groups. Although both patient groups exhibited significantly higher CDI scores than healthy controls, depressive symptom severity did not differ significantly between them, whereas serum nesfatin-1 levels remained significantly elevated in adolescents with comorbid NSSI. This interpretation was further supported by the ANCOVA findings, which demonstrated that the between-group differences in serum nesfatin-1 levels remained significant after adjustment for depressive symptom severity, indicating that the elevated nesfatin-1 levels observed in adolescents with comorbid NSSI are not explained solely by depressive symptom burden. Since the assessment of depressive disorders still relies predominantly on subjective symptom measures, the identification of objective biological correlates may contribute to a more comprehensive understanding of clinical heterogeneity in adolescent depression [39]. In the present study, ROC analyses demonstrated statistically significant discriminative ability of serum nesfatin-1 between the patient groups and healthy controls. In addition, nesfatin-1 exhibited fair discriminatory ability in distinguishing adolescents with MDD+NSSI from those with MDD alone. Although this finding suggests that nesfatin-1 may capture neurobiological differences beyond depressive symptom severity alone, its discriminatory performance was modest and should be interpreted cautiously. In addition, nesfatin-1 levels were not significantly associated with the functional dimensions of NSSI as assessed by the ISAS. This finding suggests that nesfatin-1 levels may not be closely associated with the specific motivational functions underlying NSSI, a pattern also observed with other biological markers of NSSI [3]. However, the relatively limited sample size of the MDD+NSSI group may have reduced statistical power to detect subtle associations across ISAS functional subdomains.

The present findings may also have important clinical implications. Despite comparable depressive symptom severity between the two patient groups, adolescents with comorbid NSSI exhibited significantly higher nesfatin-1 levels, suggesting that nesfatin-1 may reflect neurobiological heterogeneity within adolescent depression beyond symptom-based clinical assessment alone. Given that NSSI represents a significant clinical risk factor in adolescent depression, identification of biological processes associated with self-injurious behavior may contribute to improved understanding of risk profiles in depressed adolescents. Although the present findings do not support the standalone clinical use of serum nesfatin-1, they suggest that stress-related neuroendocrine markers may contribute to a better understanding of the biological heterogeneity of adolescent depression and NSSI. Future longitudinal studies are needed to determine whether nesfatin-1 provides incremental value beyond established clinical assessment methods.

### Study limitations and strengths

This study has several limitations. First, the cross-sectional design does not allow for the determination of causal relationships between the studied variables. In addition, the study was conducted at a single clinical center with a relatively limited sample size, which restricts the generalizability of the findings. The exclusion of adolescents with common psychiatric comorbidities, including ADHD, bipolar disorder, autism spectrum disorder, psychotic disorders, and substance use disorders, enhanced the internal validity of the study by reducing potential confounding. However, this approach may limit the generalizability of the findings, as many adolescents with major depressive disorder in routine clinical practice present with one or more psychiatric comorbidities. Therefore, multicenter studies with larger samples would be valuable for confirming and generalizing the results. Although the ROC curve analyses demonstrated encouraging discriminative performance for serum nesfatin-1, the relatively small sample size and the absence of internal validation procedures may limit the stability and generalizability of the reported diagnostic accuracy estimates. Therefore, these findings should be considered preliminary and require replication and external validation in larger, independent cohorts. The predominance of female participants in all three groups limits the generalizability of the findings to male adolescents. Additionally, the small number of male participants and the marked imbalance in sex distribution limited our ability to perform meaningful sex-specific analyses. Future studies with larger and more sex-balanced samples are needed to examine potential sex-specific effects on nesfatin-1 levels in adolescents with depression and NSSI. Serum nesfatin-1 levels may not fully reflect central nervous system nesfatin-1 concentrations, as the relationship between peripheral and central nesfatin-1 remains to be clarified. Biological factors such as participants’ dietary habits and hormonal cycles, which may influence nesfatin-1 levels, could not be controlled in the present study.

Despite these limitations, to the best of our knowledge, this is the first study evaluating the potential applicability of serum nesfatin-1 levels in predicting and assessing NSSI and depression severity in adolescents. One of the major strengths of the study is the use of a semi-structured diagnostic interview in addition to comprehensive psychiatric evaluation, which enhanced diagnostic accuracy. The inclusion of participants who were not using psychotropic or other medications eliminated potential pharmacological confounders and allowed for a more accurate assessment of nesfatin-1 levels. Evaluation of body mass index and the exclusion of individuals with metabolic syndrome minimized the potential influence of metabolic factors on nesfatin-1 levels. Considering the possible effects of circadian rhythms on nesfatin-1 levels, all blood samples were collected in the morning under fasting conditions and at a single time point, providing important methodological standardization. Additionally, the duplicate analysis of patients’ samples strengthened the reliability of laboratory measurements.

## Conclusions

The findings of this study indicate that serum nesfatin-1 levels are elevated in adolescents with MDD and are particularly increased in adolescents with comorbid NSSI. Notably, nesfatin-1 levels differentiated adolescents with MDD+NSSI from those with MDD alone despite comparable depressive symptom severity, suggesting that nesfatin-1 may be associated with neurobiological processes associated with self-injurious behavior beyond depressive symptoms alone. These findings highlight the potential value of biological markers in characterizing clinical heterogeneity within adolescent depression. Future longitudinal studies are needed to examine the trajectory of nesfatin-1 over time and its relationship with the course of NSSI.

## Data Availability

The datasets used and/or analyzed during the current study are available from the corresponding author upon reasonable request.

## Abbreviations

AUC: Area Under the Curve
BMI: Body Mass Index
CDI: Children’s Depression Inventory
CRH: Corticotropin-Releasing Hormone
DSM-5: Diagnostic and Statistical Manual of Mental Disorders, Fifth Edition
ELISA: Enzyme-Linked Immunosorbent Assay
EOS: Endogenous Opioid System
HPA: Hypothalamic–Pituitary–Adrenal
ISAS: Inventory of Statements About Self-Injury
K-SADS-PL: Schedule for Affective Disorders and Schizophrenia for School-Age Children – Present and Lifetime Version
MDD: Major Depressive Disorder
NSSI: Non-Suicidal Self-Injury
NUCB2: Nucleobindin-2
ROC: Receiver Operating Characteristic
